# How repeatable is the Environmental Impact Classification of Alien Taxa (EICAT)? Comparing independent global impact assessments of amphibians

**DOI:** 10.1002/ece3.2877

**Published:** 2017-03-19

**Authors:** Sabrina Kumschick, G. John Measey, Giovanni Vimercati, F. Andre de Villiers, Mohlamatsane M. Mokhatla, Sarah J. Davies, Corey J. Thorp, Alexander D. Rebelo, Tim M. Blackburn, Fred Kraus

**Affiliations:** ^1^ Centre for Invasion BiologyDepartment of Botany & Zoology, Stellenbosch UniversityMatielandSouth Africa; ^2^ Invasive Species ProgrammeSouth African National Biodiversity InstituteKirstenbosch National Botanical GardensClaremontSouth Africa; ^3^ Department of Genetics, Evolution & EnvironmentCentre for Biodiversity & Environment ResearchUniversity College LondonLondonUK; ^4^ Institute of ZoologyZoological Society of LondonRegent’s Park, LondonNW1 4RYUK; ^5^ Department of Ecology and Evolutionary BiologyUniversity of MichiganAnn ArborMIUSA

**Keywords:** alien species, biological invasions, impact scoring, listing, management, policy making, prioritization

## Abstract

The magnitude of impacts some alien species cause to native environments makes them targets for regulation and management. However, which species to target is not always clear, and comparisons of a wide variety of impacts are necessary. Impact scoring systems can aid management prioritization of alien species. For such tools to be objective, they need to be robust to assessor bias. Here, we assess the newly proposed Environmental Impact Classification for Alien Taxa (EICAT) used for amphibians and test how outcomes differ between assessors. Two independent assessments were made by Kraus (Annual Review of Ecology Evolution and Systematics, 46, 2015, 75‐97) and Kumschick et al. (Neobiota, 33, 2017, 53‐66), including independent literature searches for impact records. Most of the differences between these two classifications can be attributed to different literature search strategies used with only one‐third of the combined number of references shared between both studies. For the commonly assessed species, the classification of maximum impacts for most species is similar between assessors, but there are differences in the more detailed assessments. We clarify one specific issue resulting from different interpretations of EICAT, namely the practical interpretation and assigning of disease impacts in the absence of direct evidence of transmission from alien to native species. The differences between assessments outlined here cannot be attributed to features of the scheme. Reporting bias should be avoided by assessing all alien species rather than only the seemingly high‐impacting ones, which also improves the utility of the data for management and prioritization for future research. Furthermore, assessments of the same taxon by various assessors and a structured review process for assessments, as proposed by Hawkins et al. (*Diversity and Distributions*, 21, 2015, 1360), can ensure that biases can be avoided and all important literature is included.

## 1 Introduction

Species are being moved beyond the natural limits of their native ranges at a staggering rate. Some of these species (here termed alien species) have environmental impacts in the locations to which they are introduced, and such impacts are the main reason why aliens are a cause for concern. Alien species can have a diverse array of impacts, and the ways these impacts are quantified are themselves highly varied (Kumschick et al., 2015). As a result, impact scoring and classification systems are increasing in importance for invasion science and alien species management. Such systems aim to make highly diverse data on impacts comparable between species, and therefore allow patterns, trends, and potential predictors of impact to be analyzed quantitatively (e.g., Evans, Kumschick, Dyer, & Blackburn, 2014; Kumschick, Bacher, & Blackburn, 2013). They can play a crucial role in informing and guiding management decisions and creating lists of alien species with quantified impacts (e.g., Kumschick, Blackburn, & Richardson, 2016).

One of the recently developed impact scoring systems for alien species is the Environmental Impact Classification of Alien Taxa (EICAT; Blackburn et al., 2014; Hawkins et al., 2015). EICAT has been proposed to be the official classification system for alien species environmental impacts under the umbrella of the International Union for Conservation of Nature (IUCN), similar to the Red List for extinction threat (IUCN, 2012), and as one of the three essential variables necessary to monitor biological invasions, along with occupancy and alien status (Latombe et al., 2016). EICAT is based on published evidence of impact, overcoming concerns about subjectivity and knowledge bias in expert‐opinion‐based assessments and listing (e.g., Evans, Kumschick, & Blackburn, 2016; Kumschick et al., 2016). Furthermore, EICAT includes a mechanism for assigning confidence estimates in its assessments, which is important for identifying areas of uncertainty in current information, and for communicating results to stakeholders (Blackburn et al., 2014; Hawkins et al., 2015; Kumschick et al., 2017).

In any scoring system, it is important that the implementation of the method is clear and explicit enough to reduce assessor bias. Different scores may arise if the methodological formulation of the scoring system is unclear, the formulation is misinterpreted by some users, or differences in assessor background influence application of the system (Regan, Colyvan, & Burgman, 2002). For the Australian weed risk assessment—one of the most often applied and tested risk assessments for alien plants (e.g., Gassó, Basnou, & Vilà, 2010; Gordon, Onderdick, Fox, & Stocker, 2008; Kumschick & Richardson, 2013; Nishida et al., 2009; Pheloung, Williams, & Halloy, 1999)—clear guidelines were developed to counteract potential sources of bias (Gordon et al., 2010). However, it is still not always possible for different assessors to reach consensus on the outcomes of the assessments (c.f., discussion in Gordon et al., 2016). Due to its global relevance as a potential IUCN classification system for alien taxa, extensive guidelines were also developed for the EICAT scheme by Hawkins et al. (2015). In order for such guidelines to be most effective, it is important for them to address potential sources of bias in the application of the scheme. In this study, we therefore compare and contrast two classifications of the environmental impacts of alien amphibians conducted by independent parties, both using the EICAT scheme (Blackburn et al., 2014). One assessment was made before the guidelines were published (Kraus, 2015), the other one closely followed the guidelines (Kumschick et al., 2017). As both parties independently collected literature as well as performed the assessment, we can compare not only the outcomes of the classifications, but also the influence of the literature used and the underlying search effort on that outcome. We identified two main sources of potential bias, namely (1) differences in interpretation of (a) mechanisms and (b) magnitude (classifications) of impacts; and (2) differences in the literature used due to (a) different study aims or (b) different search strategies.

## 2 Methods

Two independent assessments of the environmental impact of alien amphibians worldwide were performed using the EICAT, by Kraus (2015) and Kumschick et al. (2017). The assessment by Kraus was intended as a general review of the primary literature on alien amphibian (and reptile) impacts, whereas the study by Kumschick et al. was aimed at comparing two scoring systems, and was initially set up to compare impacts between amphibians and other taxa (Measey et al., 2016). Given these different goals, the information search strategy and reporting of results differed slightly between the two applications, as outlined in Table 1, but in general terms the same classification system for impact was used by both parties.

**Table 1:** Differences in methodology applied in the two impact scoring studies

	Kraus	Kumschick et al.
Search terms	Literature from before 2007 was extracted from Kraus (2009). That list was updated up to late 2014 using Zoological Records searches limited to the years 2007–2014 with assorted combinations of search terms like: “alien species” or “invasive species” plus “impact” done for various taxonomic names such as “frog”, “amphibian”, etc.	Literature from before 2007 was extracted from Kraus (2009). Additionally, literature up to August 2015 was searched using each species’ scientific name (current and previous taxonomic iterations) in Web of Science and on Google Scholar. The results were filtered manually for relevant data on impacts by selecting publications according to the information provided in titles and abstracts, and by scanning the selection in more detail. References cited within the selected publications were screened and included as appropriate, as was gray literature. This was supplemented by more specific searches for the species name and the name of each country (according to Kraus, 2009) in which it is known to be alien. Only the primary source of information or study regarding the impacts was included on the score sheet
Magnitude	Only species for which impacts MO or higher were expected or found given the search strategy outlined above; species with lower impacts or no reports with medium or high confidence (see below) were excluded	All impacts found ranging from MC to MV were recorded (according to Hawkins et al., 2015)
Confidence	A confidence rating was not explicitly included, but only reports with medium to high confidence were used according to the assessor's interpretation	Low, medium, or high confidence (according to Hawkins et al., 2015) was attached to every single impact record, as well as the final classification per species
Initial number of species assessed	Not specified due to nature of search strategy	105 (all alien species listed in Kraus, 2009 with at least one established population plus few additional according to IUCN Red List)
Expertise on taxon	All assessments made by a single assessor with long‐term expertise on taxon	Assessments made by a team, some of whom were not experts in the taxon

The assessment by Kumschick et al. involved explicitly searching for impacts of all alien amphibian species listed by Kraus (2009) that have at least one established population. These were supplemented with two records from the IUCN Red List using searches for extralimital species (*Bombina orientalis* and *Ingerophrynus biporcatus*) (Kumschick et al., 2017). All other species are assumed to be No Alien Populations (NA) under the EICAT classification scheme (Hawkins et al., 2015). Kraus did not search for literature on each individual species, but used more general search terms due to the goal of his assessment (a general review of the literature on alien amphibian impacts of moderate or large magnitude), supplemented with more targeted searches on several species already known to have impacts (Table 1). Kraus’ assessment therefore did not include all the alien species assessed by Kumschick et al. but only those species for which he could find moderate or higher impacts with his search strategy. It was thus left unremarked whether species not assessed by Kraus fell into lower‐impact categories, or were Data Deficient (DD) under the EICAT classification scheme (Hawkins et al., 2015).

EICAT classifies alien species according to the magnitude of their impacts under a set of twelve impact mechanisms. The mechanisms are outlined in the Global Invasive Species Database (www.iucngisd.org/gisd/) and are as follows: (1) competition; (2) predation; (3) hybridization; (4) transmission of diseases to native taxa; (5) parasitism; (6) poisoning/toxicity; (7) bio‐fouling; (8) grazing/herbivory/browsing; (9) chemical; (10) physical or (11) structural impact on ecosystem; (12) interaction with other alien species. The magnitude of impact then allows species to be classified into the following categories (for this study, we follow the updated terminology in Hawkins et al., 2015): Minimal Concern (MC)—impact on individuals of at least one native taxon demonstrated, but no effect on fitness reported; Minor (MN)—reducing the fitness of individuals of one or more native taxa; Moderate (MO)—impact on populations of at least one native taxon; Major (MR)—impact on a native community that is reversible; and Massive (MV)—irreversible community‐level changes. Species for which alien populations are known, but no data on impact were found despite a standardized search, are classified as DD. Species without known alien populations based on Kraus (2009) and IUCN Red List were classified as NA.

EICAT classifications are based on evidence provided in the published and gray literatures, which were searched as described in Table 1. Each classification should be accompanied by a confidence score based on the availability and quality of the data underlying the assessment. In this study, Kumschick et al. attached a confidence score according to Hawkins et al. (2015) (low, medium, high; based on data quality, agreement between sources, and scale), whereas Kraus only included references he considered to be of medium to high confidence without reference to Hawkins et al. (2015). Confidence levels could therefore not be compared between the two studies. Each report on impact was classified separately into one of the five categories outlined above (MC to MV), and a summary classification was produced for each species after all individual reports were assessed. This summary classification used here consists of the highest category found per species, and the mechanisms through which this impact was caused. More detailed information on the classification process is described in Blackburn et al. (2014) and Hawkins et al. (2015).

## 3 Results

### 3.1 References used

**Figure 1:**
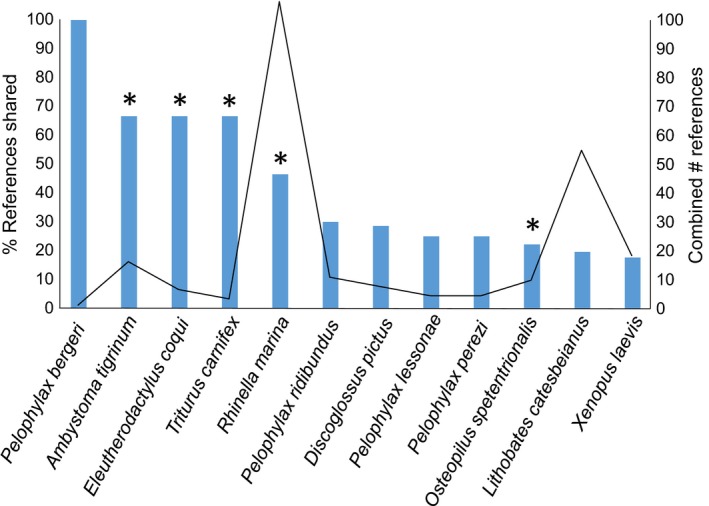
The percentage of references shared (blue bars) and the combined number of references (solid line) used by the two studies per species. Species marked with * were classified the same in both studies

The assessment by Kraus was based on 199 references that included information on the environmental impacts of 15 alien amphibian species, at an average of 13.3 studies per species. The assessment by Kumschick et al. is based on more references (242) for more species (39), but a lower average number of references per species (5.9). The difference in average arises because Kraus's assessment only considered impacts for species having at least some higher impacts, and there tend to more references for amphibian species with higher impacts. However, the relationship between the number of references used and impact magnitude turns out not to be significant (Kumschick et al., 2017). Only one‐third of the combined number of references used are shared between the studies (86 of 267). Generally, the species for which 45% or more of the references were shared between both studies led to the same overall classification (i.e., magnitude; Figure 1), with the exception of *Pelophylax bergeri* where the same (1) reference was interpreted differently in the two assessments. Nevertheless, in two cases the same classification was given based on a largely (*Osteopilus septentrionalis*) or completely (*Pelophylax bedriagae*) different set of references, however both relying on publications by the same authors and therefore similar studies.

### 3.2 Classifications (overall impact magnitude)

The impact classifications assigned to alien amphibian species by both assessments are shown in Table 2. Of the 15 species for which Kraus reported impacts, four had a highest classification of MV, 10 of MR, and one of MO. Of the 39 species for which Kumschick et al. reported impacts, four were MV, five were MR, seven were MO, 19 were MN, and four were MC. A further 66 species with alien populations were classified by Kumschick et al. as DD. Thirteen species were explicitly classified in both assessments (two species assessed by Kraus, *Pelophylax kurtmuelleri* and *Pelophylax esculentus* were excluded by Kumschick et al. due to uncertainty regarding their status as separate species, see, e.g., Akın et al., 2010). Impact classifications were the same in terms of maximum magnitude for five of these 13 commonly assessed species (Table 2). For another six species, the impact classification differed by one category (e.g., MV vs. MR for *Xenopus laevis*; Table 2). However, the assessments for two species (*Discoglossus pictus* and *Pelophylax lessonae*) differed markedly, being considered as only MN by Kumschick et al. (Table 2). Kraus and Kumschick et al. therefore concur in categorizing 11 amphibian species as of moderate to massive impact (Table 2). A further five species were classified in the higher‐impact categories (four as MO, one as MR) by Kumschick et al. but not by Kraus (Table 2). For all of these species, three or fewer papers related to impact were found, and the confidence of these classifications was rated by Kumschick et al. as “low” (except for *Pelophylax nigromaculatus*, rated “high”).

**Table 2:** Main results of the two independent assessments

		Kumschick et al.					
		MV	MR	MO	MN	MC	DD
Kraus	MV	*Ambystoma tigrinum*	*Lithobates catesbeianus* Pelophylax ridibundus *Xenopus laevis*				
	MR	*Pelophylax bergeri*	*Osteopilus septentrionalis* *Rana bedriagae* Rhinella marina *Triturus carnifex*	*Pelophylax perezi*	*Discoglossus pictus* *Pelophylax lessonae*		
	MO			*Eleutherodactylus coqui*			
	Not assessed		*Duttaphrynus melanostictus*	*Sclerophrys gutturalis* Hyla meridionalis Pelophylax nigromaculatus *Rana aurora*	*Rhinella jimi* Eleutherodactylus johnstonei Fejervarya cancrivora Fejervarya limnocharis Hoplobatrachus rugulosus Hoplobatrachus tigerinus Hylarana guentheri Lithobates berlandieri Litoria ewingii Litoria fallax Microhyla pulchra Pipa parva Plethodon jordani Pleurodema brachyops Polypedates megacephalus Scinax quinquefasciatus *Scinax ruber*	*Ichthyosaura alpestrisEleutherodactylus cystignathoidesEleutherodactylus planirostris* *Strongylopus grayii*	*Alytes obstetricans* Ambystoma laterale Sclerophrys regularis Anaxyrus americanus Aneides vagrans Bombina bombina Bombina orientalis Bombina variegata Bufo bufo Bufo gargarizans *Bufo japonicus* *Bufo mauritanicus* Dendrobates auratus Desmognathus fuscus Desmognathus quadramaculatus Duttaphrynus dhufarensis Eleutherodactylus antillensis Eleutherodactylus martinicensis Euphlyctis ehrenbergii Eurycea cirrigera Fejervarya kawamurai Fejervarya sakishimensis Gastrophryne carolinensis Glandirana rugosa Hyla cinerea Hyla intermedia Hyla japonica Hyla squirella Ingerophrynus biporcatus Kaloula pulchra Limnodynastes dorsalis Limnodynastes tasmaniensis *Lissotriton montandoni*
							*Lissotriton vulgaris* Lithobates clamitans Lithobates grylio Lithobates pipiens Lithobates septentrionalis Lithobates sphenocephala Litoria aurea Litoria cyclorhyncha Litoria ewingii Litoria raniformis Microhyla ornata Necturus maculosus Pelophylax porosus Pelophylax saharicus Physalaemus pustulosus Pipa carvalhoi Plethodon cinereus Plethodon shenandoah Polypedates leucomystax Pristimantis unistrigatus Proteus anguinus Pseudacris regilla Ptychadena mascareniensis Rana draytonii Rana sylvatica Rana temporaria Rhacophorus arboreus Rhacophorus viridis Scinax x‐signatus Speleomantes ambrosii Speleomantes italicus Speleomantes strinatii *Triturus marmoratus*

### 3.3 Species–mechanism combinations

A total of 43 species–mechanism combinations were found across the two studies for the 13 amphibian species assessed by both as having impacts. For each of these species–mechanism combinations, an impact magnitude (classification) was provided by one or both studies (Appendix S1). In eight cases, the two studies assigned the same magnitude to a specific species–mechanism combination. Of the 35 cases of difference, ten could be attributed to (1) differing interpretations of the classification scheme, namely mechanisms (six cases) and magnitude (four cases). In the majority of cases where differences were found, these could be attributed to (2) different references included (25 cases), either due to the varying study aims (seven cases; Appendix S1), that is, Kraus not including impacts lower than MO, or due to the different search strategies used (18 cases).

## 4 Discussion

The opportunity provided by two temporally coincident assessments of the environmental impacts of alien amphibians using the recently developed EICAT scheme has allowed us to assess the comparability of independent applications of this scheme and to explore reasons for differences in outcome. The outcomes of the two assessments were frequently different, but many of the differences can be attributed to different aims of the two studies rather than to features of the EICAT scheme itself. Furthermore, one assessment was performed before the guidelines (Hawkins et al., 2015) were published (Kraus), the other one (Kumschick et al.) afterward.

An obvious difference between the two studies was that Kumschick et al. provided assessments for more than twice as many amphibian species as did Kraus (39 vs. 15). This difference arose as a result of the underlying aims of each. Kraus was only interested in species with well‐supported and higher (MO to MV in the EICAT scheme) impacts because his wider aim was to review the impacts of alien amphibians, rather than to compare all amphibians in terms of their impacts. In contrast, Kumschick et al.'s aim was to classify all established alien amphibian species globally in terms of their impacts, to allow comparison of environmental impacts within and between taxa.

More interesting in terms of the EICAT methodology are differences in the categorizations resulting from the independent applications of the scheme to the subset of species reported in both studies, and to the impact categories considered in both studies. In this regard, we found different results in about half of the 13 species common to both assessments. Moreover, only 11 of the 18 species classified in the MO, MR or MV categories across both studies combined were common to both. Taken at face value, this might suggest a relatively high error rate in assigning species to impact categories. However, we think that this conclusion cannot be validly drawn from these results, for two reasons.

First, because only three of the impact categories in the EICAT scheme (MO, MR, and MV) were considered in both studies, the extent of congruence between studies may be underestimated. Kumschick et al. identified 105 amphibian species with alien populations, of which 66 were DD, and 23 assigned to impact categories not considered by Kraus. It is unknown how Kraus would have assigned most of these species using EICAT, which constitute the great majority of amphibians with alien populations. At the very least, we know he considered the majority of these species to be DD, MC, or MN, as do Kumschick et al., such that the true congruence between studies may be higher than it appears.

Second, Kraus excluded evidence of impacts that he considered to be of low confidence (the revised descriptors of confidence in Hawkins et al. (2015) were not available to him). We do not know how Kraus's classifications of these 23 additional species may have been altered by including studies with low confidence. Additional studies would not have led to lower‐impact classifications for any species, as lower‐quality data on lower impacts will not outweigh higher‐quality data identifying higher impacts in the EICAT methodology. Allowing lower‐confidence data could have resulted in higher‐impact classifications by Kraus for some species, as was the case for the seven species Kumschick et al. scored as MO or MR largely on the basis of low‐confidence data. There may thus be data legitimately to assign these species to higher EICAT categories if incorporated, increasing the overlap between the sets of higher‐impact species in the Kraus and Kumschick et al. subsets. Also of relevance in this regard, Evans et al. (2016) showed that the confidence score was positively related to impact magnitude in a global EICAT assessment for alien birds. The acceptance of low‐quality data by Kraus may not have led to many amphibian species being elevated under his scoring system, maintaining the overlap between those species he did not categorize and the MC and MN species of Kumschick et al. Generally, classification should be based on the best available evidence even if this is poor, leading to low‐confidence ratings. The main aim of incorporating confidence levels is for the low‐confidence assessments to highlight the need for more research. We do not suggest down‐rating species into lower‐impact classifications based on lower confidence, unless more research shows that a certain classification was not justified.

These issues of comparability between the Kraus and Kumschick et al. assessments highlight the importance of applying EICAT systematically to all species in a taxon in order to build a global database of alien species impacts. EICAT can be used to answer various research questions (Blackburn et al., 2014), and the selection of species or the approaches for its use might differ for various goals. However, for its use as an official tool to classify all alien species under the same framework, similarly to the Red List, EICAT does not aim to produce a list of the “worst” or most highly impacting invaders, but to provide a database of alien species in general, together with the evidence for their impacts, or the lack thereof. A key advantage of the Kumschick et al. approach is that it differentiates between species for which there is evidence of low impact (MC or MN) and species for which there is no evidence of impact (DD), despite an extensive, standardized literature search. It is widely acknowledged that not all alien species cause negative impacts, but only for a few is this lack of damage studied and demonstrated. By reporting assessments for all species, it is possible to identify where evidence of impact is of poor quality, and hence where research effort might usefully be targeted to improve our understanding of impacts. For example, only for eight species did Kumschick et al. consider their impact classification to be of high confidence, and Kraus considered only 13 species to meet high‐ or medium‐confidence levels (a more detailed assessment of differences in confidence was not possible as Kraus did not use the levels as suggested in Hawkins et al., 2015). It also highlights the many species for which no evidence of impact has even been sought (DD). This needs to be taken into account not only when comparing results between species, but also when taking management decisions and putting restrictions into place regarding species. It has been recognized that decisions need to be taken regardless of uncertainty, but this needs to be acknowledged and the sources ranked accordingly (Regan et al., 2005). Reporting assessments for all species will also help us to understand whether evidence of higher impacts by some species in the future arises from genuine change in impact status or from new evidence of preexisting impacts (Hawkins et al., 2015). Such information is important in a number of contexts, including the use of EICAT as an indicator of biodiversity change, and evidence of the success of policy changes or mitigation measures.

The differences observed between the Kraus and Kumschick et al. classifications appear not to arise from differences in the effort of their respective literature searches, but rather from the differences in the set of references included. Kumschick et al. based their assessment on more references overall, but for more species. The overlap between references included in both studies is surprisingly small (Figure 1), and the number of references used on the other hand is similar: Kumschick et al. found 207 references for the 13 species in common between their and Kraus's assessments, versus 199 references used by Kraus for the 15 species he assessed. Of these 207 references, 195–197 were available to Kraus, given that his literature review ended in September 2014, and 10–12 were published subsequently. The difference in the references used can most likely be attributed to the rather different literature search strategies adopted. Thus, Kumschick et al. searched through all the literature on every established alien species, with no further restrictions in search terms, whereas Kraus looked for literature on amphibians in general, but restricted the results by adding additional search terms like “alien” or “impact,” and excluding dietary studies (Table 1). The differences in the reference base used for the assessments seem to be the primary reason for the different classifications (Appendix S1). Furthermore, there is an obvious association between the different search strategies and the number of species assessed, as Kraus only classified 15 species with higher impact, while Kumschick et al. classified 105 species, including species with low or no recorded impacts (and 16 of higher impact). These differences are again the result of the different study aims, however.

More generally, the literature available to different assessors may be expected to vary based on their location and affiliation. Access to the primary scientific literature can be a problem for assessors outside the university system, whereas older (non‐digitized) literature and small‐circulation regional journals are not always readily available even within the university system. Access to gray literature is certain to be highly context dependent. For example, undergraduate theses, technical reports, and impact survey reports may be more readily available to assessors located in or near the location invaded by an alien species, yielding data sources that would not be available to a library and not be picked up by internet‐based searches. Such issues may be compounded for invasions in developing countries where publication levels are lower, access to the primary literature harder, and language problems more likely (i.e., many papers published in local languages). These potential limitations may have played a small role in the particular cases studied here. For example, Kraus did not have access to the report used by Kumschick et al. for *Sclerophrys gutturalis*; conversely, Kumschick et al. did not have access to some of the unpublished agency reports providing some of the strongest evidence of predation impacts from *Lithobates catesbeianus*.

We suggest that given the limited overlap between references included in the two studies (Figure 1), differences in interpreting the EICAT criteria are not the primary reason for differences in the classifications by Kraus and Kumschick et al. The limited cases where differences could be attributed to different interpretation may have arisen because the extensive criteria and guidelines developed by Hawkins et al. (2015) for implementing EICAT were not available to Kraus. These criteria were specifically produced to eliminate ambiguities, and to ensure as far as possible that all classifications were consistent and comparable. We suspect that the use of these in both assessments would have increased the congruence in outcomes. For example, *D. pictus* was classified as MR by Kraus on the basis of a study that found that the native amphibian community was more structured (comparing checkerboard scores in invaded vs. noninvaded plots) where it was not invaded by this species, and that native species’ populations were partially displaced from some breeding ponds (Richter‐Boix et al., 2013). According to the EICAT guidelines (Hawkins et al., 2015), impacts are only scored as MR if they engender a *compositional* change to community structure. Richter‐Boix et al. do not directly report any population declines or loss of species from the invaded community, which resulted in a classification of MN for *D. pictus* by Kumschick et al., but the interpretation of this piece of evidence is not unambiguous.

Nevertheless, differences between assessors are still possible even with thorough and detailed guidelines. One of the main issues encountered in this study was interpretation of the irreversibility of impact. In four cases, species were classified as MV in one assessment and MR in the other, based largely on the same references: A key difference between MR and MV impacts is whether or not they are reversible. The guidelines by Hawkins et al. (2015) devote a paragraph to the definition of irreversibility, defining it to mean “that there is evidence that removal of the alien would not result in [a return] to the pre‐invasion state.” An obvious example is if invasion results in extinction. They do however also allow for irreversibility “in practice,” where “the effort or cost required is so large that it would not happen, even if in theory it might be possible.” This aspect is more open to interpretation.

A further example of the difficulties of interpreting EICAT criteria which was uncovered when comparing the two independent studies is the transmission of diseases from alien amphibians to native species. Amphibian disease, especially chytridiomycosis and its causative agent *Batrachochytrium dendrobatidis (Bd)*, has become a major research area in recent years, following massive enigmatic declines of native amphibians on several continents (Berger et al., 2016). Alien amphibians (specifically *X. laevis*) were argued to have caused the global chytridiomycosis pandemic (Weldon, du Preez, Hyatt, Muller, & Speare, 2004). Yet, despite data corroborating the arrival of disease with trade, and the existence of diseased animals in the trade (van Sittert & Measey, 2016), very few studies to date have demonstrated a link between alien populations and the transmission of disease to native amphibians. For this reason, Kumschick et al. scored impacts on the basis of the ability of alien amphibian populations to act as reservoirs for *Bd*, and not on the basis of them having introduced the disease which then caused decline in native species. Kraus scored the impact of certain frogs which carry *Bd* as MV in recognition of a combination of factors: the irrefutably dire (often irreversible) impacts created by the disease, reports of the disease in alien populations of these frogs (Garner et al., 2006; Hanselmann et al., 2004), first discovery of *Bd* in Britain in a recently established population of *L. catesbeianus* (Cunningham et al., 2005; Fisher & Garner, 2007), frequent asymptomatic infection of *L. catesbeianus* and *X. laevis* (Daszak et al., 2004; Mazzoni et al., 2003; Weldon et al., 2004) that makes each species effective disease vectors, and temporal correlation of the spread of *Bd* with the wide dissemination of *L. catesbeianus* and *X. laevis* in the 20th century. Hence, it seemed likely that both species have contributed to the spread of *Bd* (Fisher & Garner, 2007), although Kraus (2015) noted that they were not the sole vectors responsible. This difference in expert views highlights the difference between different types of evidence of impact, which are discussed by Hawkins et al. (2015).

Overall, there is no study which has *shown* the transmission of chytridiomycosis from alien to native amphibians, but it has been *inferred* from the combination of several studies. It is important to note such possibilities for high impacts when connecting evidence from various studies, but such classifications should generally not be rated with high confidence (see also the EICAT guidelines provided by Hawkins et al., 2015). Where we do not have direct evidence for transmission of the disease from alien to native species, we suggest that the following pieces of evidence are both needed in order to classify species as MO or higher for impact from disease transmission: (1) The disease agent has shown to be highly devastating to native species (see also disease agents EICAT profile, see below); (2) the alien species is a host of the disease agent in the same time and space as the native population occurs. If these conditions are met at a certain location, no direct evidence for disease transmission is needed. If one of these factors is missing, the alien species should get a “red flag” indicating that more research is needed. Ideally, we would also be interested to know whether the disease agent arrived with the alien, or whether it had an effect on the native community before the alien arrived. However, these aspects cannot be retrospectively assessed and are therefore virtually impossible to study when the invasion has already occurred.

Often we find evidence for the alien species being a host of a (more or less devastating) disease (e.g., Fisher & Garner, 2007), and in some cases, spread of the disease with the alien host is studied (e.g., Hanselmann et al., 2004; Jancovich et al., 2005). In these cases, we suggest that impacts through transmission of diseases under EICAT should be scored as MN. It can in most cases not be scored MC as the guidelines state “The alien taxon is not a host of diseases…” (Hawkins et al., 2015), unless the disease or parasite carried by the alien was not found in the native species (e.g., Dubey & Shine, 2008). Furthermore, one needs to distinguish between the impact of the disease itself and the impact of the host. We suggest performing an EICAT assessment separately for the disease agent and linking this to the assessment of disease transmission of the host. This can also be important for management as removing a host from an area might not solve the disease problem itself if the disease agent is already widespread in the native community or if it is not reliant on the alien host.

Generally, differences in interpretation such as the one identified here for disease impacts highlight a feature of the EICAT scheme not implemented in the two assessments compared here, but which has the specific aim to ensure consistency in classifications: namely, a review process for each assessment (Hawkins et al., 2015). At the time of writing, processes are underway to have EICAT adopted as the formal mechanism by which IUCN categorizes the environmental impact of alien species. As for the Red List, one element of this is the requirement that all assessments face independent peer review prior to being formally accepted as the classification for a species. This aims to check that the criteria have been applied correctly, that the evidence has been interpreted correctly in respect of the criteria, and that the supporting evidence is sufficient to justify the resulting classification (cf. IUCN, 2012). Reviews are intended to help issues of interpretation such as those related to reversibility of impacts. Ultimately, the EICAT process will result in a single, widely accepted classification for each species, which should inform analysis and management of alien species impacts.

## 5 Conclusions

Although two independent assessments of the environmental impacts of alien amphibians produce somewhat different categorizations using the same impact scheme, these differences cannot be attributed to features of the scheme. Rather, differences in the literature used, study aim, approach to low‐quality data, and interpretation play a role, with the first three of these being most important in this case. The differences in scoring between the two assessments emphasize the need for a thorough data search strategy. Species specific searches and assessments are recommended to ensure that all important references are covered, and assessors should not focus on (seemingly) high‐impacting species as this will lead to a reporting bias and reduced utility of the data for both management and further studies related to impact magnitude. The differences in assessments also highlight the need for consistency checks regarding the scoring methodology and a review of the classification in general. The clear guidelines and framework developed for EICAT (Hawkins et al., 2015) should ensure that most of these biases can be avoided in future assessments. Furthermore, a process of peer review of assessments would reduce variance in assessment outcomes, for example, by reducing the likelihood that key sources of impact evidence are missed.

## Conflict of Interest

None declared.

## Author Contributions

SK and GJM conceived the ideas; SK, GJM, and TMB led the writing; SK, GJM, GV, FAV, MMM, SJD, CJT, ADR, and FK collected the data; all authors contributed to the writing and revision of the manuscript.
