# Supplementary material for: How repeatable is the Environmental Impact Classification of Alien Taxa (EICAT)? Comparing independent global impact assessments of amphibians
Source: Ecol Evol. 2017 Mar 19;7(8):2661–70. doi: 10.1002/ece3.2877 (PMC5395449; doi:10.1002/ece3.2877)
Supplement: Supplementary file 1 [file ECE3-7-2661-s001.docx]

Supplementary Material

Appendix S1: A list of the impact mechanisms recorded for each of the 13 amphibian species assessed in both studies, together with explanations for observed differences in classification between the two studies. Difference categories are: 1) differences in interpretation of a) mechanisms and b) magnitude (classifications) of impacts; and 2) differences in the literature used due to a) different study aims or b) different search strategies.

| **Species** | **Mechanism** | **Magnitude Kraus** | **Magnitude Kumschick et al.** | **Higher magnitude** | **Comments** | **References** | **Difference category** |
| --- | --- | --- | --- | --- | --- | --- | --- |
| *Ambystoma tigrinum* | Predation and/or Competition | Major | - | Kraus | no real difference, both study found both types of impacts. general agreement on category | 67% shared | 1a |
|  | Disease transmission | Moderate | Minor | Kraus+1 | disagreement with classifying disease transmission, general agreement on category | 67% shared | 1b |
|  | Hybridization | Massive | Massive | same | some differences in lower scoring mechanisms, but general agreement on category | 67% shared | na |
|  | Predation | Moderate | Moderate | same | some differences in lower scoring mechanisms, but general agreement on category | 67% shared | na |
| *Discoglossus pictus* | Competition | Major | Minor | Kraus+2 | different interpretation of same study. community structure affected, but no species lost | 29% shared | 1b |
|  | Predation | - | Minor | Kumschick et al. | Kraus generally only included impacts for species having MO or higher magnitude | 29% shared | 2a |
| *Eleutherodactylus coqui* | Disease transmission | - | Minimal Concern | Kumschick et al. | Kraus excluded Minimal Concern literature, but general agreement on category | 67% shared | 2a |
|  | Parasitism | - | Minimal Concern | Kumschick et al. | Kraus did not take into account Minimal Concern literature, but general agreement on category. Parasitism interpreted differently by the two studies | 67% shared | 2a |
|  | Predation | Moderate | Moderate | same | Kraus excluded Minimal Concern literature, but general agreement on category | 67% shared | na |
| *Lithobates catesbeianus* | Predation and/or Competition | Moderate | Major | Kumschick et al.+1 | some mechanisms scored higher by the one, some by the other, but general agreement on category | 20% shared | 2b |
|  | Disease transmission | Massive | Major | Kraus+1 | some mechanisms scored higher by the one, some by the other, but general agreement on final classification | 20% shared | 2b |
|  | Competition | Massive | Major | Kraus+1 | some mechanisms scored higher by the one, some by the other, but general agreement on category | 20% shared | 2b |
|  | Herbivory | Moderate | Major | Kumschick et al.+1 | some mechanisms scored higher by the one, some by the other, but general agreement on category | 20% shared | 2b |
|  | Predation | Major | Major | same |  | 20% shared | na |
| *Osteopilus septentrionalis* | Predation | - | Major | Kumschick et al. | same categories, differences in some scores, but general agreement | 22% shared | 2b |
|  | Predation and/or Competition | Moderate | Major | Kumschick et al.+1 | same categories, differences in some scores, but general agreement | 22% shared | 2b |
|  | Competition | Major | Minor | Kraus+2 | same categories, differences in some scores, but general agreement | 22% shared | 2b |
| *Pelophylax bergeri* | Competition | Major | - | Kraus | Kumschick et al. found no competition impact; different interpretation of same study | 100% shared; both based on same reference (1) | 1a |
|  | Hybridization | Major | Massive | Kumschick et al.+1 | different interpretation of same study | 100% shared; both based on same reference (1) | 1b |
| *Pelophylax lessonae* | Hybridization | Major | Minor | Kraus+2 | different references | 25% sharing; one study overlapping | 2b |
|  | Disease transmission | - | Minimal Concern | Kumschick et al. | Kraus excluded Minimal Concern literature | 25% sharing; one study overlapping | 2a |
| *Pelophylax perezi* | Predation and/or Competition | Major | Moderate | Kraus+1 | Kraus could not distinguish between the two mechanisms and scored higher, while Kumschick et al. scored competition and predation separately | 25% sharing; one study overlapping | 2b |
|  | Competition | - | Moderate | Kumschick et al. | Kumschick et al. scored competition and predation separately | 25% sharing; one study overlapping | 2b |
|  | Predation | - | Moderate | Kumschick et al. | Kumschick et al. scored competition and predation separately | 25% sharing; one study overlapping | 2b |
|  | Disease transmission | - | Minimal Concern | Kumschick et al. | Kraus excluded Minimal Concern literature | 25% sharing; one study overlapping | 2a |
| *Pelophylax ridibundus* | Hybridization and/or competition | Massive | - | Kraus | Kumschick et al. did not score competition impact, but both agree on hybridisation impact | 30% sharing; Kraus extra 2 references | 1a |
|  | Competition | Major | - | Kraus | Kumschick et al. did not score competition impact (literature missed) | 30% sharing; Kraus extra 2 references | 1a |
|  | Disease transmission | - | Minimal Concern | Kumschick et al. | Kraus excluded Minimal Concern literature | 30% sharing; Kraus extra 2 references | 2a |
|  | Predation | - | Minor | Kumschick et al. | Kraus generally only included impacts MO or higher | 30% sharing; Kraus extra 2 references | 2a |
|  | Hybridization | Major | Major | same |  | 30% sharing; Kraus extra 2 references | na |
| *Rana bedriagae* | Hybridization | Major | Major | same |  | no shared reference, but same classification | na |
| *Rhinella marina* | Interaction | - | Minor | Kumschick et al. | Kraus excluded impacts <MO | 46% sharing; both > 90 references | 2b |
|  | Competition | - | Moderate | Kumschick et al. | not taken into account by Kraus due to low confidence in competition studies | 46% sharing; both > 90 references | 2b |
|  | Chemical, structural and physical | - | Minor | Kumschick et al. | Kraus excluded impacts <MO | 46% sharing; both > 90 references | 2b |
|  | Disease transmission | Moderate | Moderate | same |  | 46% sharing; both > 90 references | 2b |
|  | Predation | Major | Moderate | Kraus+1 | Different magnitude in some categories, but general agreement | 46% sharing; both > 90 references | 2b |
|  | Poisoning | Major | Major | same |  | 46% sharing; both > 90 references | na |
| *Triturus carnifex* | Competition | - | Minimal Concern | Kumschick et al. | Kraus excluded Minimal Concern literature | 67% shared | 2a |
|  | Hybridization | Major | Major | same |  | 67% shared | na |
| *Xenopus laevis* | Parasitism | - | Minor | Kumschick et al. | Parasitism interpreted differently by the two studies | 18% shared; Kumschick et al. twice as many references | 2b |
|  | Predation and/or Competition | Major | - | Kraus | Kumschick et al. did not score competition impact based on study conclusion, but same predation magnitude | 18% shared; Kumschick et al. twice as many references | 1a |
|  | Predation | - | Major | Kumschick et al. | Kumschick et al. scored predation separately, but same magnitude | 18% shared; Kumschick et al. twice as many references | 1a |
|  | Disease transmission | Massive | Minor | Kraus+3 | disagreement with classifying disease transmission | 18% shared; Kumschick et al. twice as many references | 1b |
